# Catalogue of Lewis's Medical and Scientific Library

**Published:** 1888-09

**Authors:** 


*Catalogue of Lewis's Medical and Scientific Library.* Pp. 267. London: H. K. LEWIS. 1888.

This catalogue, "revised to the Midsummer of 1887, and including a classified index of subjects, with the names of those authors who have treated upon them," may be regarded as a fairly complete list of the most modern medical and scientific works; and as such will be found of value, not only to the discriminating purchaser of books, but to the reader and student. At a glance he can see what men have written on any subject; a further easy reference gives the full title and date of the publication; while last, but not least, the price is uniformly appended.

